# 

**Published:** 1891-04

**Authors:** 


A Compend of Human Anatomy, including the Anatomy of the Viscera. By Samuel O. L. Potter, M. A., M. D., Professor of Theory and Practice of Medicine in the Cooper Medical College of San Francisco; author of a Hand-book of Materia Medica, Pharmacy and Therapeutics, etc., etc., etc.; late A. A. Surgeon U. S. Army. Fifth edition, revised and enlarged, with 117 wood engravings; also an appendix containing numerous tables and sixteen lithographic plates on the nerves and arteries. The Quiz Compend Series. Philadelphia: P. Blakiston, Son & Co., No. 1012 Walnut street. 1890.

This popular "quiz compend" has reached the dignity of a fifth edition. An appendix of forty-three pages, contains original tables and plates of the arteries, the cranial and spinal nerves and plexuses and the lymphatic nervous system. Now and then an inaccuracy creeps in, as for instance in plate four, page 251, where the gluteal artery is represented as considerably smaller than the internal pudic, sciatic or obturator arteries, whereas it is the largest branch of the internal iliac, and should be so represented. The student will find the book convenient and useful in preparing for examination, and the doctor will get in it, in a moment, almost any point upon which he has become rusty. J. P.

